# PIASγ Enhanced SUMO-2 Modification of Nurr1 Activation-Function-1 Domain Limits Nurr1 Transcriptional Synergy

**DOI:** 10.1371/journal.pone.0055035

**Published:** 2013-01-24

**Authors:** Cristian Arredondo, Marcelo Orellana, Andrea Vecchiola, Luis Alberto Pereira, Leopoldo Galdames, María Estela Andrés

**Affiliations:** Millennium Nucleus in Stress and Addiction, Department of Cellular and Molecular Biology, Faculty of Biological Sciences, Pontificia Universidad Católica de Chile, Santiago, Chile; National University of Singapore, Singapore

## Abstract

Nurr1 (NR4A2) is a transcription factor that belongs to the orphan NR4A group of the nuclear receptor superfamily. Nurr1 plays key roles in the origin and maintenance of midbrain dopamine neurons, and peripheral inflammatory processes. PIASγ, a SUMO-E3 ligase, represses Nurr1 transcriptional activity. We report that Nurr1 is SUMOylated by SUMO-2 in the lysine 91 located in the transcriptional activation function 1 domain of Nurr1. Nurr1 SUMOylation by SUMO-2 is markedly facilitated by overexpressing wild type PIASγ, but not by a mutant form of PIASγ lacking its first LXXLL motif (PIASγmut1). This PIASγmut1 is also unable to interact with Nurr1 and to repress Nurr1 transcriptional activity. Interestingly, the mutant PIASγC342A that lacks SUMO ligase activity is still able to significantly repress Nurr1-dependent transcriptional activity, but not to enhance Nurr1 SUMOylation. A SUMOylation-deficient Nurr1 mutant displays higher transcriptional activity than the wild type Nurr1 only in promoters harboring more than one Nurr1 response element. Furthermore, lysine 91, the major target of Nurr1 SUMOylation is contained in a canonical synergy control motif, indicating that SUMO-2 posttranslational modification of Nurr1 regulates its transcriptional synergy in complex promoters. In conclusion, PIASγ can exert two types of negative regulations over Nurr1. On one hand, PIASγ limits Nurr1 transactivation in complex promoters by SUMOylating its lysine 91. On the other hand, PIASγ fully represses Nurr1 transactivation through a direct interaction, independently of its E3-ligase activity.

## Introduction

Nurr1 (NR4A2) is a transcription factor with several functions, but highlights for its key role inducing and maintaining midbrain dopamine neurons of the mammalian central nervous system [1]. Nurr1 together with Nur77 (NR4A1, NGFI-B) and Nor1 (NR4A3) conform the NR4A group of the nuclear receptor superfamily [2]. Nurr1 shares with the other NR4A members a highly conserved structural organization. This structure consists of an almost identical DNA-binding domain (DBD); a moderately conserved C-terminal region, which encloses both the ligand-binding domain (LBD) and the transcriptional activation function-2 (AF-2); and the N-terminal region containing the AF-1, which is the most divergent domain [3]. Nurr1 binds DNA as monomer to the NGFI-B response element (NBRE, 5′ AAAGGTCA 3′) and as homo or heterodimer with Nur77 to the NurRE elements. Furthermore, Nurr1 and Nur77 can form heterodimers with the retinoid X receptor binding to DR5 elements [1]. Traditionally, nuclear receptors regulate transcription in a ligand-dependent manner, but the three members of the NR4A subfamily are classified as “orphan” because they are not associated with ligands [3]. Structural studies have shown that the LBD domain of Nurr1 lacks the cavity to accommodate a ligand, and its AF-2 adopts naturally a stable transcriptional active conformation [4]. In addition, current data indicates that Nurr1 is not regulated by traditional transcriptional coactivators [5]. Since Nurr1 is not regulated by endogenous ligands, post-translational modifications are one of the most significant mechanisms to regulate Nurr1 transcriptional activity.

Previously, we suggested that Nurr1 is SUMOylated since we probed that Nurr1 interacts with PIASγ a SUMO-E3 ligase [6]–[7] and that this interaction inhibits Nurr1-dependent transcriptional activity [8]. SUMOylation is a post-translational modification of proteins that involves the attachment of the small ubiquitin-like modifier (SUMO) peptide to the target protein. In mammals there are four SUMO peptides: SUMO-1, SUMO-2, SUMO-3 and SUMO-4. The SUMO modification process requires the action of an E1 activating enzyme (SAE1/SAE2), the E2 conjugation enzyme (Ubc9) and an E3-ligase enzyme [9]. The conjugation of SUMO to proteins is through an isopeptide bond between the C-terminus of SUMO and a ε-amino group of a lysine residue in the target protein; this lysine residue is often located in a consensus sequence composed of a characteristic ΨKXE motif [9]. SUMOylation is a reversible process, in which the de-SUMOylation is exerted by SUMO-specific proteases (SENP) [10]. SUMOylation of transcription factors regulate their half-life, the subcellular location and the transcriptional activity, among other features [11]–[12]. Interestingly, SUMOylation of several transcription factors as the glucocorticoids, androgen and estrogen nuclear receptors, restricts their transcriptional activity in promoters with several response elements arranged in tandem [13]. This SUMOylation occurs in lysines overlapping with a synergy control (SC) motif [14]. Here, we report that Nurr1 is SUMOylated by SUMO-2 at the lysine 91 located in a functional SC motif. Thus, SUMOylation of Nurr1 in the lysine 91 restricts its transcriptional activity in promoters with more than one response element. We show also that PIASγ enhances Nurr1 SUMOylation on lysine 91. Thus, we conclude that PIASγ exert two mechanisms of repression over Nurr1 transactivity, one dependent and other independent of Nurr1 SUMOylation.

## Materials and Methods

### Plasmid constructions

The hemagglutinin (HA)-Nurr1 expression vector (pCGN-Nurr1) encoding full-length rat Nurr1, the mutant HA-Nurr1-K91R and Myc-Nurr1_363–598_ were described previously [8]. Point mutation HA-Nurr1-K74R was generated by overlapping PCR and the mutated fragment was cloned into pCGN-Nurr1 using XbaI/PstI sites. Point mutations of HA-Nurr1-K558R and PIASγC342A were generated by site-directed mutagenesis, using mutagenic primers and pfu polymerase in a PCR reaction followed by DpnI digestion for 1 hour. To generate HA-Nurr1ΔAF-2 (residues 1–353), HA-Nurr1 was cut with BamHI to get rid of the fragment 354–598 and re-ligated. HA-Nurr1ΔAF-2-K91R was similarly generated from HA-Nurr1-K91R. HA-Nurr1ΔAF-1 (residues 262–598) was obtained by PCR using specific primers spanning the coding region for amino acids 262–598 and cloned in frame with HA in pCGN plasmid. pcDNA3.1-HA-Nurr1 expression vector was generated from pCGN-Nurr1 using PCR to generate an HA-Nurr1 fragment that was inserted in pcDNA3.1+ within EcoRV/NotI sites. GST-PIASγ was generated by cloning the human cDNA of PIASγ coding for amino acids 1–158 in frame with gluthation-S-transferase in the pGEX-4T3 vector. GST-PIASγmut1 y GST-PIASγmut2 were generated by overlapping PCR using primers codifying alanine instead leucine in the sequence coding for each LXXLL (for mut1 gac-***ctt-***cag-atg-***ctc-ctg-***ggt was changed by gac-***gct-***cag-atg-***gcc-gcg-***ggt and for mut2 atg-***ctg-***gat-gag-***ctg-ctg-***aag was changed by atg-***gcg-***gat-gag-***gcg-gcg-***aag). All mutagenesis and constructs were confirmed by sequencing and correct protein expression was checked by western blot.

The reporter plasmid NBRE-3X-tk-Luciferase [5] that contains three mer NBRE elements was kindly donated by Dr. T. Perlmann (Karolinska Institute, Sweden). pcDNA3.1-HIS-SUMO-1, pcDNA3.1-HIS-SUMO-2 and pcDNA3.1-HIS-SUMO-3 were kindly donated by Dr. R. Hay (Center for Biomolecular Sciences, Scotland). 1NBRE-LUC and 3NBRELUC [15] were kindly donated by Dr. A. Winoto (University of California, Berkeley, California, USA). pcDNAPIASγ was kindly donated by Dr. F. White (Indiana University School of Medicine, Indianapolis, USA). The expression vectors for SENP1 and SENP1-dominant negative (SENP1-DN) [16] were donated by Dr. O'Hare (Marie Curie Research Institute, UK).

### Cell culture and transfections

COS-7, COS-1 and HEK293 cell lines obtained from American Type Culture Collection (ATCC) were cultured in Dulbecco's modified Eagle's medium (DMEM), supplemented with 10% fetal bovine serum (FBS) and maintained at 37°C and 5% CO2, and supplemented with 1% penicillin/streptomycin. PC12 cell line also was obtained from ATCC and cultured in DMEM supplemented with 5% FBS and 10% horse serum, and maintained in 10% CO2. Transfections were carried out using Lipofectamine2000 reagent (Invitrogen).

### Immunoprecipitation and western blot

Cells were grown and transfected in 100-mm plate and lysed with 0.9 mL of lysis buffer (50 mM Tris-HCl pH 7.5, 50 mM NaCl, 1 mM EDTA, 1% NP40), supplemented with a cocktail of protease inhibitors and 20 mM of N-ethylmaleimide. Cell extracts were immunoprecipitated as follow: pre-cleared with 10 μL of protein A/G PLUS agarose beads (Santa Cruz Biotechnology) plus 0.5 μg of preimmune mouse IgG, during 1 hour at 4°C. The extracts were recovered by centrifugation and incubated with 1 μg of specific antibody during 3 hours and then with 15 μL of protein A/G PLUS agarose beads for an additional 1.5 hours. The beads were pelleted, and washed two times with lysis buffer and two times with PBS. Finally, bound proteins were eluted by boiling in 2X Laemmli sample buffer. Immunoprecipitated proteins were fractionated by SDS-PAGE and western blots performed with a monoclonal anti-HA antibody (Covance) or a monoclonal anti-Nurr1 antibody (447C2a, Santa Cruz).

### GST-pull-down assays

GST-Pull-down assays were performed essentially as we have described [17]. Protein concentration and purity of recombinant GST-PIASγ_1–158_, wild type and mutants, were determined by densitometry on 12% poliacrilamida-SDS gel stained with coomassie blue and compared to a BSA concentration curve. All recombinants GST-PIASγ migrated at the expected molecular weight of about 46 kD (26 kD of GST and 20 kD PIASγ_1–158_) and a minor band of about 39 kD. Both bands were recognized by anti-PIASγ antibody. To test the interaction between GST-PIASγ_1–158_ (wild type and mutants) and Nurr1, whole extracts of COS-1 cells transfected with Myc-Nurr1_363–598_ were incubated with GST-PIASγ fusion proteins or GST alone (1.5 µg) bound to glutathione-agarose for 1 h at 4°C. After extensive washing, the retained proteins were eluted with sample buffer and resolved on 12% SDS-PAGE. Immunoblots were incubated with monoclonal anti-Myc (9E10).

### Mammalian reporter gene assays

Cells were sowing in 24-well plates and transfected with 100 ng of the reporter plasmid and an equivalent molar amount of expression plasmids or empty vectors (pCGN or pcDNA3.1+). Total amount of DNA (400 ng) was kept constant by adding pBluescript SR (Stratagene). A reporter gene expressing the β-galactosidase cDNA driven by the cytomegalovirus promoter was cotransfected (20 ng) in all experiments as an internal control for transfection efficiency. Cells were harvested 48 hours after transfection. Luciferase activities were normalized to the activity of the internal control β-galactosidase. Each set of experiments was performed in triplicate and repeated at least three times.

### Immunofluorescence and confocal analysis

The immunofluorescence and co-localization analysis were performed essentially as we have described [18]. Briefly, fixed and permeabilized cells were incubated with polyclonal anti-PIASγ 1/500 and monoclonal anti-HA 1/500 overnight. After exhaustive washing cells were incubated with following secondary antibodies: donkey anti-goat Alexa Fluor 488 (green) and donkey anti-mouse Alexa Fluor 594 (red). Immunofluorescence images were captured with a confocal microscope (Olympus®, Fluoview 1000). Quantification of fluorescence colocalization was done using the method described by van Steensel et al. [19]. Cross correlation function (CCF) of dual labeling images was calculated by shifting the green image with respect to the red image or vice versa.

*Statistical analyses* – Results are expressed as mean ± S.E.M. from at least three independent assays. Statistical analyses were performed using the non-parametric Mann-Whitney U test.

## Results

### Nurr1 is SUMOylated

**Figure 1:**
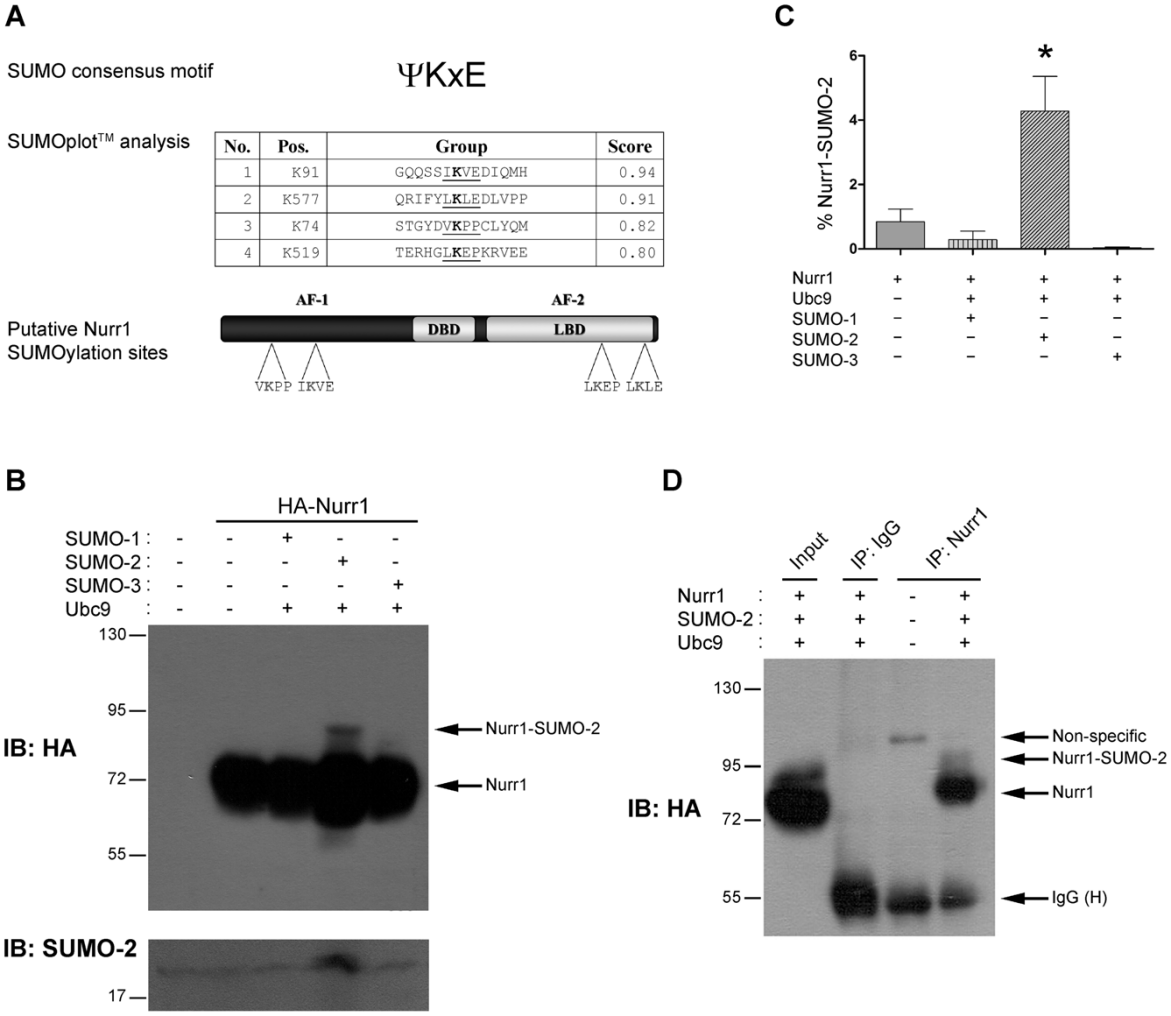
Nurr1 is SUMOylated by SUMO-2. (A) Schematic representation of Nurr1 (bottom) showing the position of four putative SUMOylation sites according with SUMOplot^TM^ software analysis (middle). The table shows the sequences of the potential SUMOylation sites of rat Nurr1 sorted from the highest score. Putative SUMO acceptor lysines (K) are highlighted, and potential SUMO sites are underlined. (B) COS-7 cells were transfected with plasmids expressing HA-Nurr1, Ubc9, and SUMO-1, SUMO-2 or SUMO-3. Cells were harvested 48 hours post-transfection and lysed directly in loading buffer containing the SUMO-isopeptidase inhibitor N-ethylmaleimide 20 mM, and fractionated in SDS-PAGE. Representative western-blot with anti-HA (upper) and anti-SUMO-2 (bottom) antibodies. (C) Quantitative densitometry analysis of Nurr1-SUMO-2 signal described in (B), using Image J software. Data correspond to the mean ± S.E.M. of 3 independent experiments for each condition. Statistical significance was estimated by the non-parametric Mann-Whitney U-test. * p<0.05 (Nurr1+Ubc9+SUMO-2 v/s Nurr1). (D) Total lysates from COS-7 cells transfected with HA-Nurr1, Ubc9 and SUMO-2 were immunoprecipitated with an anti-Nurr1 antibody or control IgG. The immunoprecipitates were analyzed in western blots with anti-HA antibody. Bands for immunoglobulin are indicated as IgG heavy chain (H).

Previously, we identified PIASγ as an interacting partner of Nurr1 [8]. This result suggested us that Nurr1 is a SUMO target protein. According with SUMOplot^TM^ software, Nurr1 has four lysines with high score to be SUMO target: lysine 91 and lysine 577 that are in canonical ΨKXE motifs, and lysines 74 and 519 located in non-canonical SUMO sequences, but showing a high score for SUMOylation (Fig. 1A). To determine if Nurr1 is a target of SUMO peptide, we overexpressed Nurr1 along with each of the SUMO peptides (SUMO-1, SUMO-2 or SUMO-3) plus Ubc9 in COS-7 cells. Immunoblotting of the cell extracts showed two immunoreactive bands, a major band of about 72-kDa corresponds to the expected unmodified HA-Nurr1 and a band of lesser intensity and more slowly migrating of about 95-kDa. This 95-kDa band is observed only in the condition when SUMO-2 and Ubc9 were overexpressed (Fig. 1B). The quantification of the 95-kDa slower migrating band indicates that only around a 3% of HA-Nurr1 is significantly SUMOylated (Fig. 1C). To further confirm that Nurr1 is a target of SUMO-2, we carried out coimmunoprecipitation assays. As shown in figure 1D, western blot with anti-HA antibody detected two bands, one corresponding to the unmodified HA-Nurr1 and a slower migrating diffuse signal specifically found in precipitates with anti-Nurr1. These results indicate that Nurr1 is a target of SUMO-2 posttranslational modification.

### Identification of major SUMO sites in Nurr1

**Figure 2:**
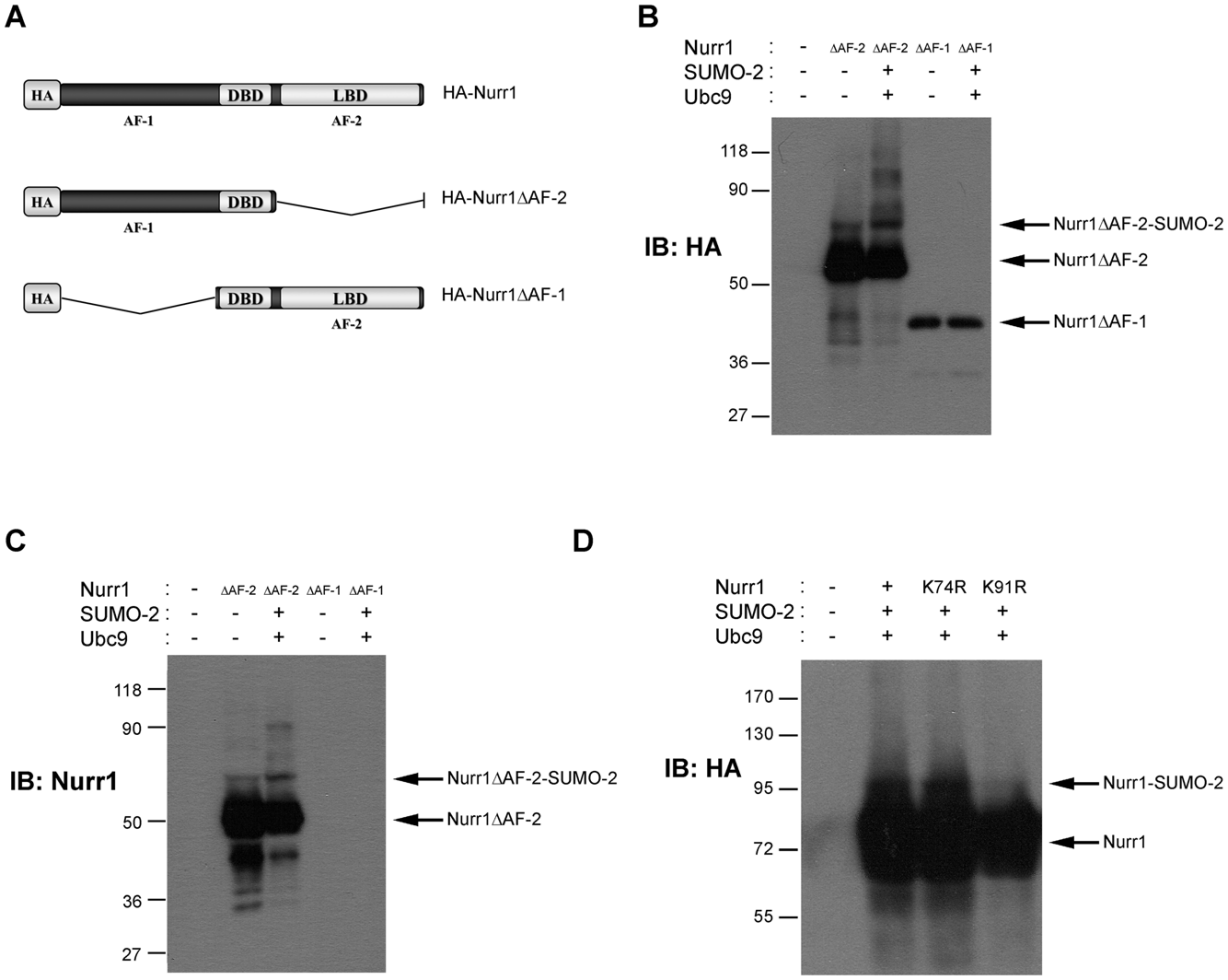
Lysine 91 is the main SUMOylation site of Nurr1. (A) Schematic representation of full-length HA-Nurr1, and truncated isoforms HA-Nurr1ΔAF-2 (amino acids 1–353) and HA-Nurr1ΔAF-1 (amino acids 262–598). (B, C) Total extracts of COS-7 cells transfected with HA-Nurr1ΔAF-2 or HA-Nurr1ΔAF-1, plus Ubc9 and SUMO-2 were fractionated in SDS-PAGE and proteins analyzed with anti-HA (B) and anti-Nurr1 (C) antibodies. (D) Total extracts of COS-7 cells transfected with wild type HA-Nurr1 or the point mutants HA-Nurr1-K74R or HA-Nurr1-K91R were fractionated in SDS-PAGE. Western blot was performed with an anti-HA antibody.

The lysines with high score for SUMOylation in Nurr1 are within the transcriptional activation domains of this transcription factor. Lysines 74 and 91 are within the AF-1 domain, while lysines 519 and 577 are within the AF-2 domain of Nurr1 (Fig. 1A). In order to determine faster the segment of Nurr1 that is target of SUMO-2 we worked with truncated forms of Nurr1 (HA-Nurr1ΔAF-1 or HA-Nurr1ΔAF-2) containing only one of the transcriptional activation domains plus the DBD (Fig. 2A). Western blot assays performed either with anti-HA (Fig. 2B) or anti-Nurr1 (Fig. 2C) showed a slower migrating band of Nurr1 only when HA-Nurr1ΔAF-2 was overexpressed (Fig. 2B, C). When HA-Nurr1ΔAF-1 was overexpressed, we detected only the band corresponding to the unmodified HA-Nurr1ΔAF-1 fragment of Nurr1 (Fig. 2B). Overexposure of this film failed to show additional bands that could suggest post-translational modification of this fragment of Nurr1 (data not shown). HA-Nurr1ΔAF-1 fragment was not detected with the anti-Nurr1 antibody used, because this antibody was raised against the N-terminal domain of Nurr1 (Fig. 2C). Together the data indicate that Nurr1 is SUMOylated by SUMO-2 in the AF-1. Therefore, we continue with lysines 74 and 91 as the putative SUMOylation targets in Nurr1. We replaced lysines 74 and 91 with arginines (Nurr1-KΔR mutants) and overexpressed each HA- epitope tagged Nurr1-KΔR mutant along with SUMO-2 and Ubc9 in COS-7 cells. Immunoblotting of extracts of cells expressing these constructs revealed that the HA-Nurr1-K91R mutant was only weakly SUMOylated, but HA-Nurr1-K74R was SUMOylated similarly to wild type HA-Nurr1 (Fig. 2D). This evidence indicates that lysine 91 is the major target of SUMO-2 in Nurr1.

### PIASγ enhances SUMOylation of Nurr1

**Figure 3:**
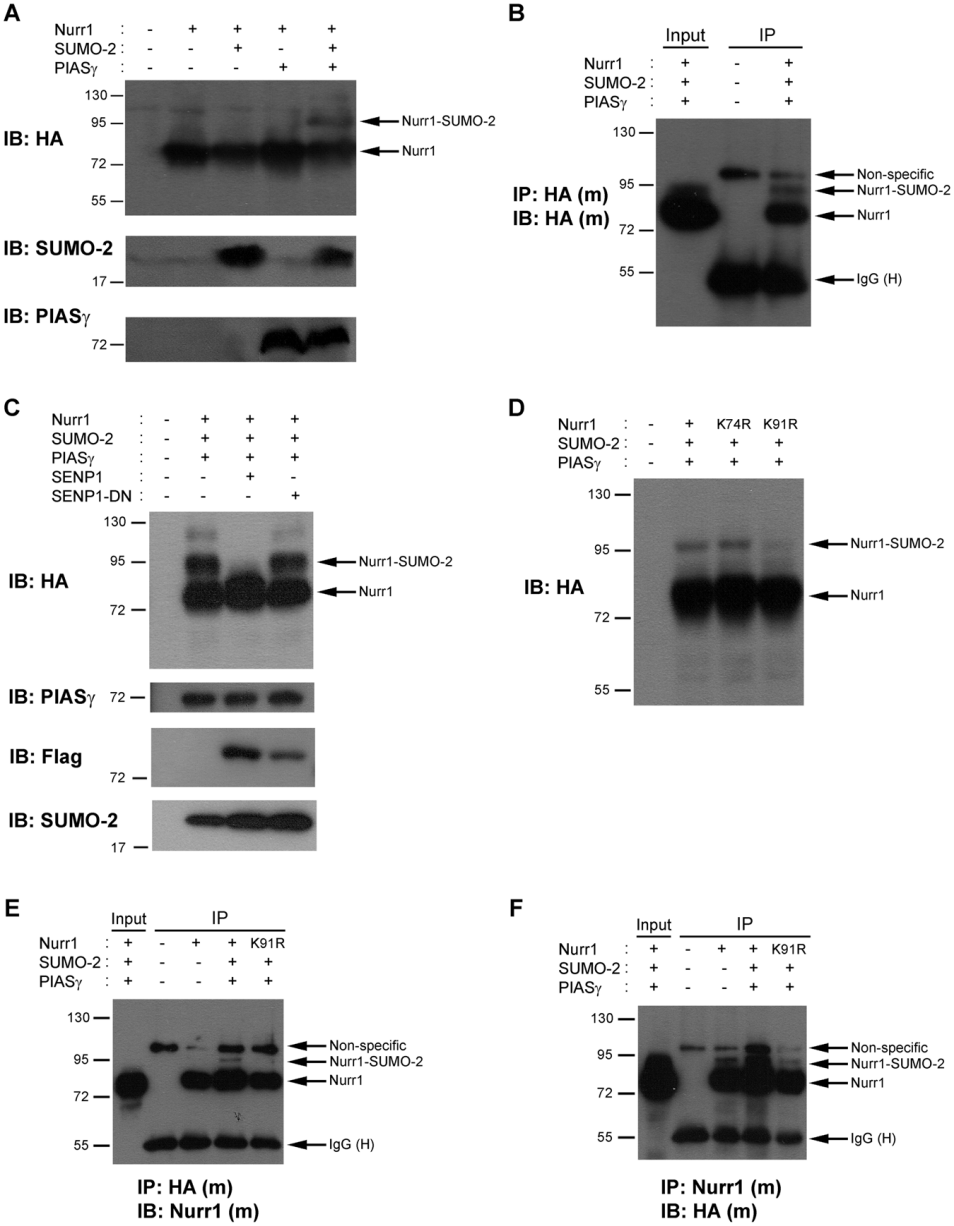
PIASγ enhances Nurr1 SUMOylation. (A) Total extracts of COS-7 cells transfected with plasmids encoding HA-Nurr1, SUMO-2 and PIASγ were fractionated in SDS-PAGE and western blots were performed with anti-HA (upper), anti-SUMO-2 (middle) and anti-PIASγ (bottom) antibodies. (B) Total extracts of COS-7 cells transfected as in (A) were immunoprecipitated and immunoblotted with a monoclonal (m) anti-HA antibody. (C) Total extracts of COS-7 cells transfected with HA-Nurr1, SUMO-2, PIASγ and Flag-SENP1 or Flag-SENP1-DN were fractionated in SDS-PAGE and western blots were performed with the indicated antibodies. (D) Total extracts of COS-7 cells transfected with HA-Nurr1 or the point mutants HA-Nurr1-K74R or HA-Nurr1-K91R were fractionated in SDS-PAGE. Western blots were performed with an anti-HA antibody. (E, F) Total extracts of COS-7 cells transfected with HA-Nurr1 or HA-Nurr1-K91R plus SUMO-2 and PIASγ were immunoprecipitated with anti-HA antibody and western blot carried out with anti-Nurr1 antibody (E) or immunoprecipitated with anti-Nurr1 antibody and western blot carried out with anti-HA antibody (F). (m): monoclonal, (H): IgG heavy chain.

Because PIASγ interacts and regulates Nurr1 [8], we tested the hypothesis that PIASγ is the SUMO-E3 ligase in Nurr1 SUMOylation process. To this end, we overexpressed PIASγ instead Ubc9, along with Nurr1 and SUMO-2 in COS-7 cells. The immunoblot of these cell extracts showed the slower 95-kDa migrating band (Fig. 3A) corresponding to Nurr1-SUMO-2. The quantification of the slower migrating band of 95-kDa indicates that about an 8.56±3.90% (p<0.03) of the total Nurr1 became SUMOylated in the presence of PIASγ. In addition, coimmunoprecipitation assays performed from extracts of COS-7 cells overexpressing PIASγ instead of Ubc9, along with SUMO-2 and HA-Nurr1 showed a clear slower migrating 95-kDa band corresponding to HA-Nurr1-SUMO-2 (Fig. 3B). The 95-kDa band was totally lost when the de-SUMOylating enzyme SENP1 was overexpressed (Fig. 3C), but not when the variant dominant negative SENP1-DN [16] was overexpressed in the same conditions (Fig. 3C). These evidences indicate that PIASγ behaves as a SUMO-E3 ligase for Nurr1 SUMOylation.

Then, we studied whether lysine 91 is also the major SUMOylation site of Nurr1 when PIASγ is overexpressed. Immunoblots showed that the slower migrating 95-kDa band is significantly decreased in cell extracts of HA-Nurr1-K91R mutant, but not in the HA-Nurr1-K74R mutant (Fig. 3D). To support further lysine 91 as the SUMO target of Nurr1, coimmunoprecipitation assays were carried out. As shown in figure 3E, immunoblotting of immunoprecipitated Nurr1 with anti-HA (Fig. 3E) or anti-Nurr1 (Fig. 3F) antibodies showed the HA-Nurr1 band and a well-defined 95-kDa slower migrating band corresponding to the SUMOylated form (Fig. 3D). The slower 95-kDa band decreased significantly in the mutant HA-Nurr1-K91R compared to wild type HA-Nurr1 immunoprecipitates (Fig. 3E, F).

**Figure 4:**
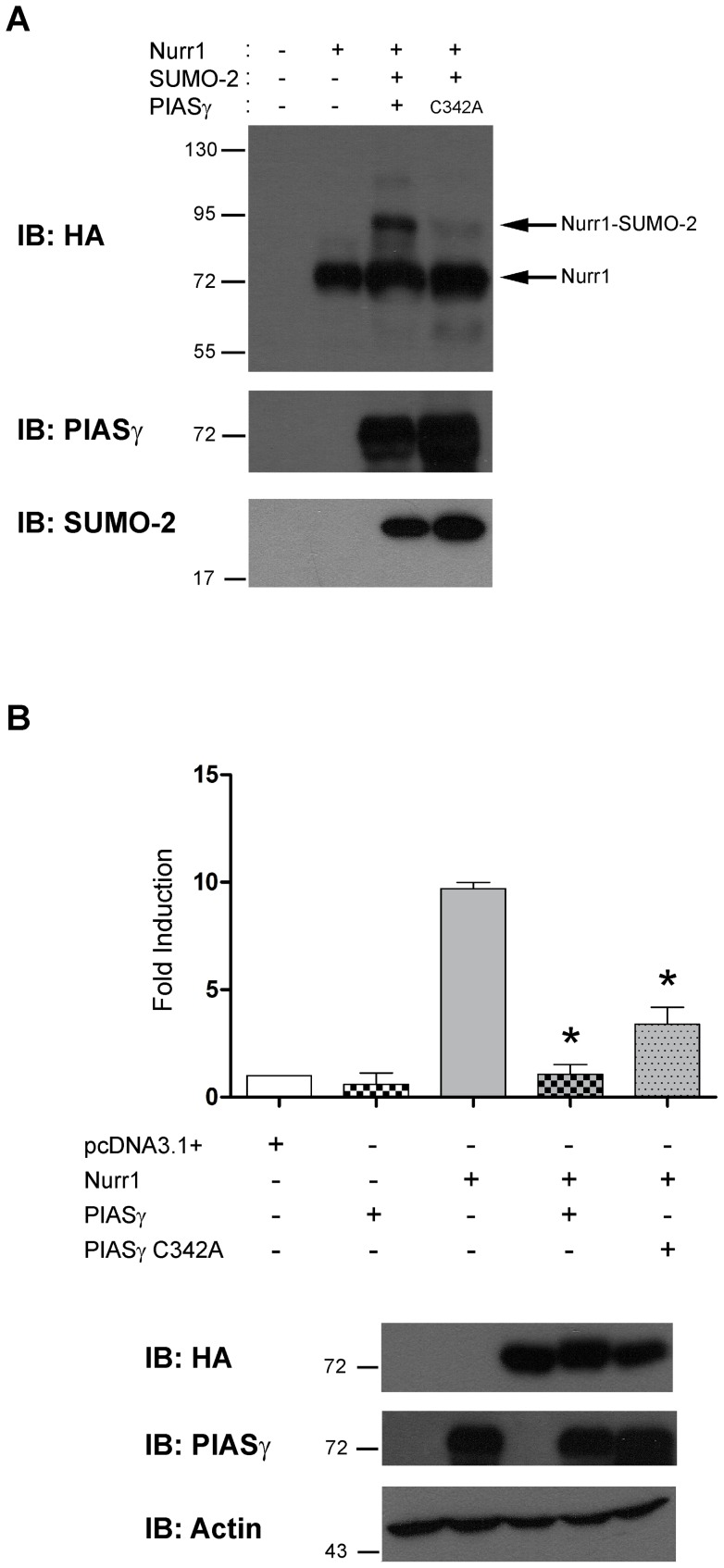
Point mutant PIASγC342A fails to SUMOylate Nurr1. (A) Total extracts of COS-7 cells transfected with HA-Nurr1, SUMO-2 and PIASγ or the point mutant PIASγC342A were fractionated in SDS-PAGE and western blot assays performed with anti-HA (upper), anti-PIASγ (middle) and anti-SUMO-2 (bottom) antibodies. (B) HEK293cells were transfected with 100 ng of NBRE-3X-tk-LUC reporter and equimolar amounts of HA-Nurr1, PIASγ or PIASγC342A. Cells were harvested 48 hours post transfection and lysates assayed for luciferase activity. Results are expressed as fold of induction related to control (pcDNA3.1+) and correspond to the mean ± S.E.M. of three independent assays performed each in triplicate. Statistical significance was estimated by the non-parametric Mann-Whitney U test. *p<0.05 (Nurr1+PIASγ v/s Nurr1) and (Nurr1+ PIASγC342A v/s Nurr1). In the bottom, western blots showing the expression of recombinant proteins and actin used as loading control.

To test whether the SUMO E3-ligase enzymatic activity of PIASγ is required to SUMOylate Nurr1, we replaced the cysteine 342 by alanine, PIASγC342A, which revokes SUMO ligase activity of PIASγ [20]. As shown in figure 4A, the mutant PIASγC342A was not able to enhance Nurr1 SUMOylation, albeit the amount of overexpressed wild type and mutant PIASγ is similar in the cell extracts (Fig. 4A). This set of experiments show that PIASγ mediates SUMO-2 conjugation on lysine 91 of Nurr1 as a SUMO-E3 ligase.

As previously mentioned, PIASγ not only interacts with Nurr1, but also inhibits its transcriptional activity [8]. Thus, we asked whether the SUMOylation activity of PIASγ is required for its inhibitory action over Nurr1. Surprisingly, the mutant PIASγC342A was still able to repress significantly Nurr1 transcriptional activity (Fig. 4B). However, the repression exerted by PIASγC342A was lower than wild type PIASγ (Fig. 4B).

**Figure 5:**
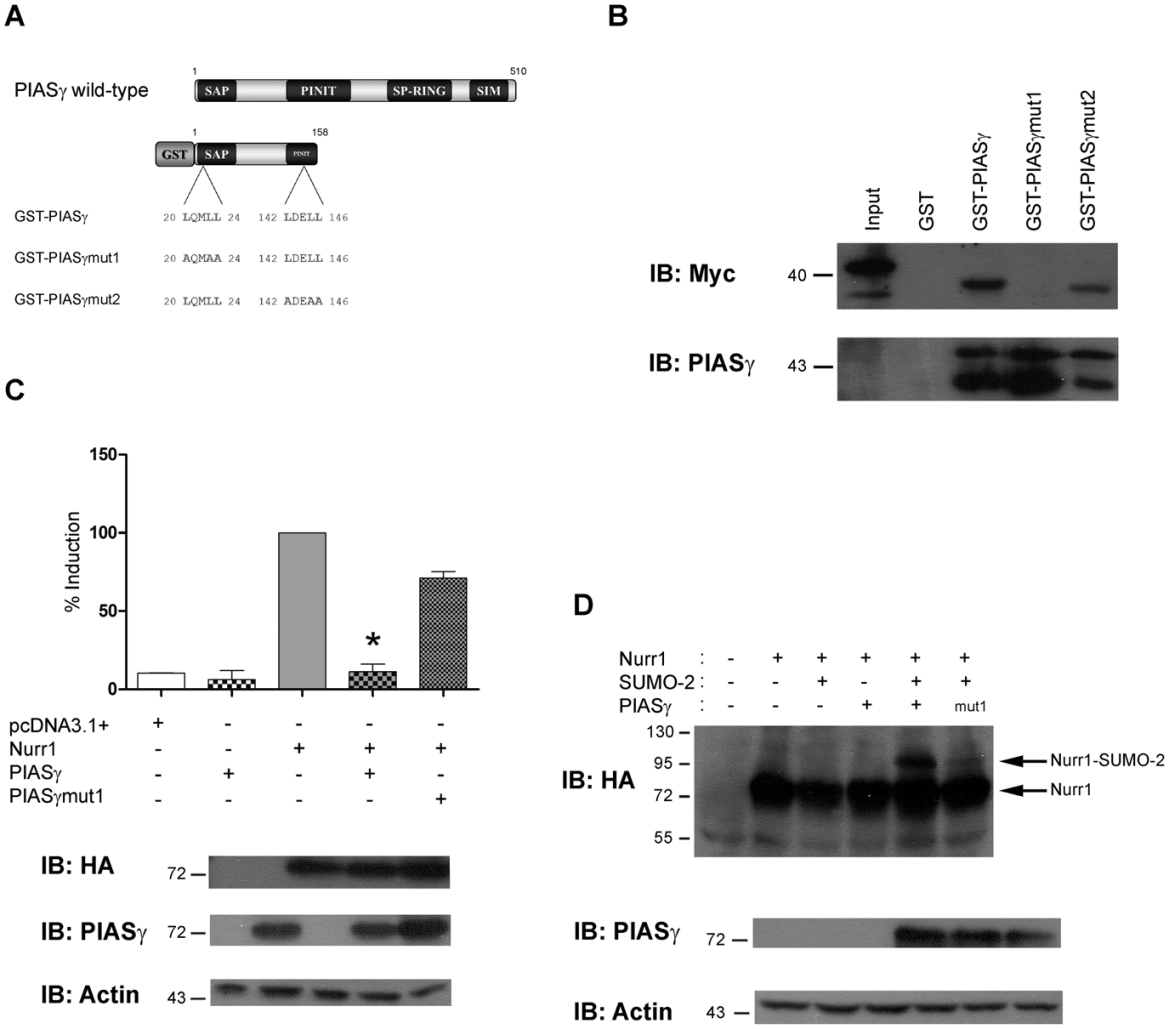
PIASγ requires the _20_LXXLL_24_ motif to interact, SUMOylate and repress Nurr1. (A) Schematic representation of full-length PIASγ indicating functional domains SAP: *Scaffold Attachment factor-A/B acinus and PIAS*; the PINIT domain; SP*-*(*Siz/PIAS*)*-*RING and SIM: *SUMO Interaction Motif*. PIASγ has two LXXLL motifs. Mutations changing leucines (L) by alanines (A) in each LXXLL domain are depicted for GST-PIASγmut1 and GST-PIASγmut2. (B) GST, GST-PIASγ, GST-PIASγmut1 and GST-PIASγmut2 retained in glutathione-agarose beads were incubated with extracts from COS-1 cells transfected with Myc-Nurr1_363–598_. Retained proteins were fractionated in SDS-PAGE and western blot developed with anti-Myc monoclonal antibody, revealing that only GST-PIASγ and GST-PIASγmut2 are able to interact with Nurr1. Recombinant GST-PIASγ proteins were equally loaded in each GST-pull down assays as shown by western blot using anti-PIASγ antibody (bottom). (C) Luciferase reporter assay showing that full-length PIASγmut1 lose repressor capacity over Nurr1 transactivity. HEK293 cells were transfected with the NBRE-3X-tk-LUC along with HA-Nurr1, PIASγ or PIASγmut1. After 48 hours, cells extracts were assayed for luciferase activity. Data are expressed as percentage of Nurr1 transactivation and correspond to the mean ± S.E.M of 4 independent experiments each performed in triplicates. Statistical significance was estimated by the non-parametric Mann-Whitney U test. *p<0.05 (Nurr1+PIASγ v/s Nurr1+PIASγmut1). In the bottom, western blots showing the expression of recombinant proteins and actin used as loading control. (D) Total extracts of COS-7 cells transfected with HA-Nurr1, SUMO-2 and PIASγ or PIASγmut1 were fractionated in SDS-PAGE and western blots performed with anti-HA, anti-PIASγ and actin (load control) antibodies.

To further characterize the role of PIASγ regulating Nurr1 SUMOylation and its transcriptional activity, we determined the domain of PIASγ needed to interact with Nurr1. As shown in figure 5A, PIASγ has two LXXLL motifs located between amino acids 20–24 and 142–146. LXXLL motifs are essential for some coregulators to interact with transcription factors [21]. Thus, we replaced leucines by alanines in each LXXLL motif (Fig. 5A) of PIASγ to learn whether any or both of these motifs mediate the interaction between Nurr1 and PIASγ. As shown in figure 5B, PIASγ encompassing the first 158 amino acids of the protein was able to retain specifically the recombinant Myc-Nurr1_363–598_ [8] in GTS-pull down assays. GTS-PIASγmut1, lacking the first LXXLL motif lost the capacity to interact with Myc-Nurr1_363–598_ (Fig. 5B); however, the GST-PIASγmut2 was able to retain it (Fig. 5B), indicating that PIASγ requires only the first _20_LXXLL_24_ motif for the interaction with Nurr1. The lack of interaction between Nurr1 and PIASγmut1 was not due to changes on the amount of recombinant GST-PIASγ constructs, since similar amount of GST-PIASγ constructs were observed with an anti-PIASγ antibody (Fig. 5B, lower panel). These results prompted us to study the effect of full-length PIASγmut1 lacking the first LXXLL motif on Nurr1 transcriptional activity. As shown in figure 5C, full-length PIASγmut1 was unable to repress Nurr1-dependent transcriptional activity, compared to the total transcriptional repression exerted by wild type PIASγ (Fig. 5C). PIASγmut1 showed similar expression than wild type PIASγ (Fig. 5C lower panel) indicating that the lack of repressive effect is due to the lack of interaction with Nurr1 and not to a wrong localization and/or lower expression. In addition, overexpression of PIASγmut1 did not enhance Nurr1 SUMOylation by SUMO-2 as compared with wild type Nurr1 (Fig. 5D). Thus, PIASγ requires its first LXXLL motif to interact, repress and SUMOylate Nurr1.

**Figure 6:**
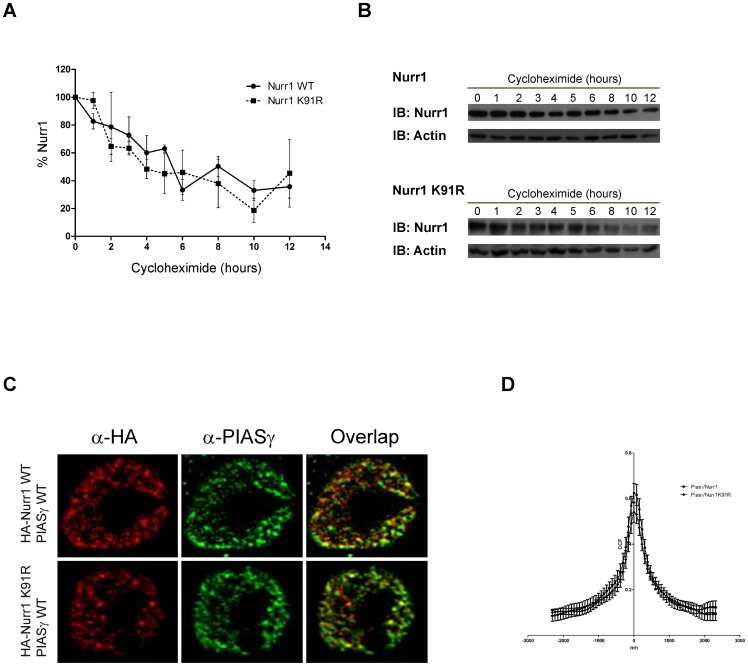
SUMOylation on lysine 91 does not modify Nurr1 half-life and location. (A) HEK293 cells were transfected with HA-Nurr1 or HA-Nurr1-K91R. Twelve hours after transfection the cells were treated with cycloheximide and harvested at the indicated hours. Total extracts were fractionated in SDS-PAGE and western blots developed with anti-HA and anti-actin (load control) antibodies. Densitometric analysis of 3 independent experiments was performed with Image J software. Data is expressed as percentage of HA-Nurr1 or HA-Nurr1-K91R expression at cero time and correspond to the mean ± S.E.M. (B) Representative western blots of HA-Nurr1 and HA-Nurr1-K91R during cycloheximide treatment. (C) PC12 cells were transfected with the indicated plasmids. Fixed cells were subjected to double immunofluorescence protocols using HA and PIASγ antibodies. Alexa 594 (red) second antibody was used to visualize HA and Alexa 488 (green) second antibody was used to visualize PIASγ. Cells were examined by deconvolution microscopy. (D) Colocalization of Nurr1 or the mutant Nurr1-K91R with PIASγ using van-Steensel analysis [19].

SUMOylation can regulate nuclear receptor localization, stability and transcriptional activity, among other functions [9] [11]–[12]. The amount of recombinant Nurr1 and Nurr1-K91R in HEK293 cell extracts observed at different time after cycloheximide treatment show that both HA-Nurr1 and the mutant HA-Nurr1-K91R have a similar half-life (Fig. 6A, B) of about six hours [22], suggesting that SUMOylation of lysine 91 is not involved in Nurr1 stability. Previously, we showed that Nurr1 and the mutant Nurr1-K91R are located in the nuclei of cells [8] suggesting that Nurr1 SUMOylation in K91 does not modify its subcellular localization. Here we show, by confocal immunofluorescence analysis, that the mutant Nurr1-K91R has a similar subnuclear localization than the wild type Nurr1 (Fig. 6C). In addition, Van Steensel coefficient quantification [19] indicates that PIASγ colocalize equally well with HA-Nurr1 and the mutant HA-Nurr1-K91R (Fig. 6D), suggesting a similar capacity of interaction, a result that reinforce the idea that PIASγ is able to repress equally well the transcriptional activity of wild type Nurr1 and the mutant Nurr1-K91R.

### SUMO consensus motif of Nurr1 overlaps with a transcriptional synergy control motif

**Figure 7:**
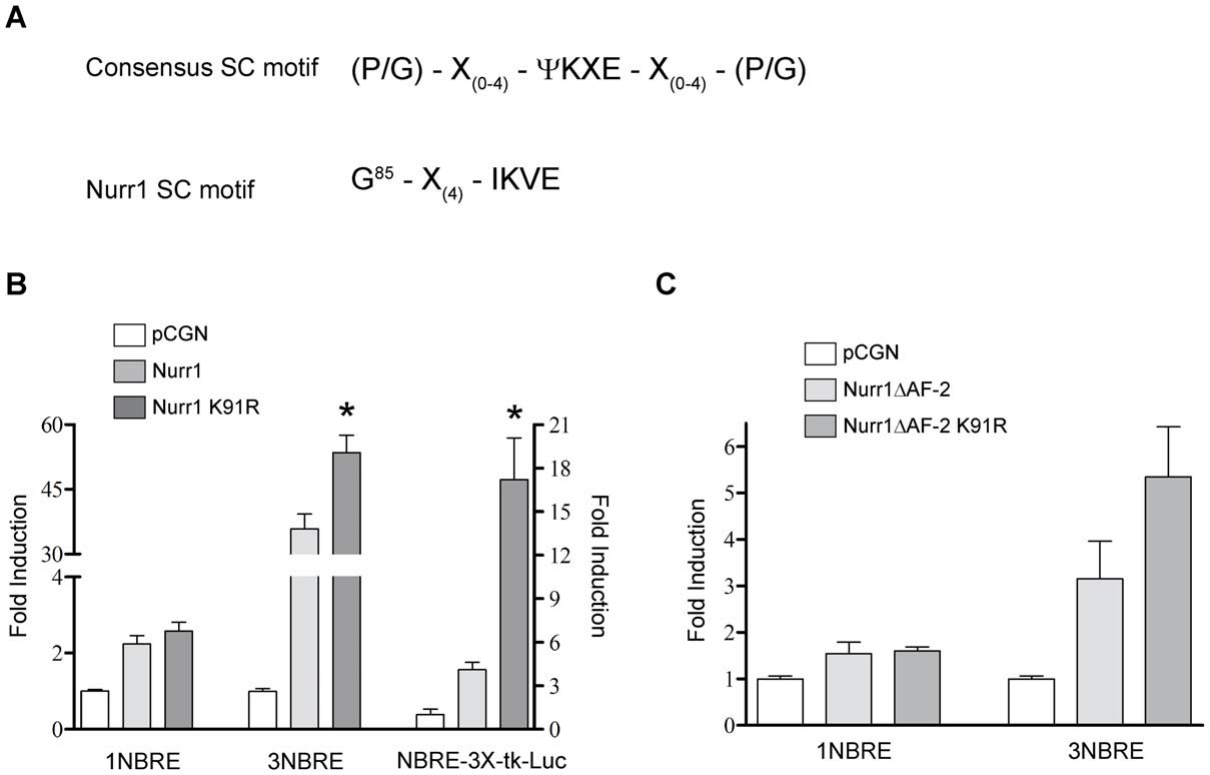
Lysine 91 of Nurr1 is in a synergy control (SC) motif. (A) Consensus SC motif [14] and putative Nurr1 SC motif. (B) HEK293 cells were transfected with 100 ng of 1NBRE-Luc (one NBRE element), 3NBRE-Luc (three NBRE elements) [15] or NBRE-3X-tk-LUC (three NBRE elements) [5] reporters and equimolar amounts of HA-Nurr1 or point mutant HA-Nurr1-K91R. Cells were harvested 48 hours post-transfection and lysates assayed for luciferase activity. Results are expressed as fold of induction related to control (pCGN, empty vector) and correspond to the mean ± S.E.M. of three independent assays performed each in triplicate. Scale in left axis is for 1NBRE-Luc and 3NBRE-Luc reporters' activity, and scale in right axis is for NBRE-3X-tk-LUC reporter activity. Statistical significance was estimated by the non-parametric Mann-Whitney U test *p<0.05 (HA-Nurr1-K91R v/s HA-Nurr1). (C) HEK293cells were transfected with 100 ng of 1NBRE-Luc or 3NBRE-Luc reporters and equimolar amounts of HA-Nurr1ΔAF-2 or point mutant HA-Nurr1ΔAF-2-K91R. Cells were harvested 48 hours post-transfection and lysate assayed for luciferase activity. Results are expressed as fold of induction related to control (pCGN) and correspond to the mean ± S.E.M. of three independent assays performed each in triplicate.

Some years ago, Iñiguez-Lluhi and Pearce [14] identified a motif in several transcription factors that restricts transcriptional activation in promoters with more than one response element for the transcription factor, denominated complex promoters. The motif was called “synergy control” (SC) and its consensus sequence includes a SUMO motif flanked by proline [14] or glycine [23] residues (Fig. 7A). Because the lysine 91 of Nurr1 lies within a putative SC motif (Fig. 7A), we tested the hypothesis that SUMOylation of lysine 91 restricts synergic Nurr1-dependent transcription. To this end, we compared the transcriptional activity of HA-Nurr1 and the mutant HA-Nurr1-K91R using luciferase reporter constructs containing one mer (1NBRE) and three mer (3NBRE; NBRE3X-tk). As expected, wild type HA-Nurr1 synergistically activated transcription from the three mer NBRE reporters, leading to more luciferase activity compared with the one mer NBRE construct (Fig. 7B). As we have previously shown [8], HA-Nurr1-K91R mutant induced a significant higher luciferase activity than wild type HA-Nurr1 using NBRE3X-tk-Luc reporter (Fig. 7B). HA-Nurr1-K91R mutant also showed a higher transcriptional activity than wild type HA-Nurr1 using another luciferase reporter that also contains three mer NBRE elements [15], but that showed a stronger induction with Nurr1 overexpression (Fig. 7B). Remarkably, HA-Nurr1-K91R showed similar transcriptional activity compared with wild type HA-Nurr1 in the reporter containing only one mer NBRE element (Fig. 7B). Similar results were obtained when we transfected the wild type and K91R mutant forms of truncated Nurr1 lacking AF-2 domain. As shown in figure 7C, similar slight transcriptional activation was induced by wild type HA-Nurr1ΔAF-2 and HA-Nurr1ΔAF-2-K91R from one mer (1NBRE) reporter, while the mutant HA-Nurr1ΔAF-2-K91R induced a higher luciferase activity than HA-Nurr1ΔAF-2 from three mer (3NBRE) reporter. These results indicate that the lysine 91 of Nurr1 is in a true SC motif, whose SUMOylation control synergic transcription activity from promoters containing multiple NBRE elements.

## Discussion

We have previously shown that PIASγ interacts and inhibits Nurr1 transcriptional activity [8], leaving as an open question whether Nurr1 is, in fact, SUMOylated. Here, we show that Nurr1 is a target of SUMO-2 peptide, and that lysine 91 is its major SUMOylation acceptor site. The results also indicate that lysine 91 of Nurr1 is within a functional SC motif. Lysine 91 in this SC motif restricts Nurr1 transcriptional activity in promoters containing more than one NBRE element. In addition, we report that PIASγ behaves as a SUMO-E3 ligase for Nurr1.

We observed a small, but significant percentage of Nurr1 SUMOylated (about 3% in the presence of Ubc9 and 8% in the presence of PIASγ) in our experimental conditions. This small amount of Nurr1 SUMOylated does not match with the 30–60% increment observed in the transcriptional activity induced by the mutant Nurr1-K91R compared with the wild type Nurr1 (Fig. 7). This paradox known as the “SUMO enigma” has been observed for several transcriptional factors, allowing the proposal that SUMOylation is required to initiate a response but not to maintain it [9] [11]–[12]. The “SUMO enigma” hypothesis [9] [11]–[12] sustains that SUMOylation is a habilitation mark to be incorporated to a transcription repressor complex, but it would be not necessary to be kept in the complex. This hypothesis is also consistent with the broad regulatory repertoire that a transcription factor can exert depending on the promoter used. For example, our data show that wild type Nurr1 induces 30 times one promoter (3NBRE), [15] and only 3 times the other promoter (NBRE-3x-tk-Luc) [5], in circumstances that both promoters have 3 in tandem NBRE elements. In addition, no differences are observed between wild type Nurr1 and K91R mutant when the promoter has only one NBRE element. By SUMO-modifying the entire pool of Nurr1, the transactivation of each promoter would be proportional to the initial transcription effect and our data suggest that this is not the case. These data suggest that Nurr1 SUMOylation should occur in a promoter dependent manner to give a fine, differential response for each case.

We found that SUMO-2 is the only SUMO peptide post-translational modifying Nurr1. We cannot exclude that Nurr1 is a target of SUMO-1 or SUMO-3 in this or other cellular contexts. However, preferential SUMOylation by one of the SUMO peptides is increasingly being reported [24]. Our results indicate that lysine 91 is the major SUMO acceptor site in Nurr1. Since a faint signal of the slower migrating 95-kDa band remains in immunoblots with the mutant K91R, it is possible that another minor SUMOylation site is present in Nurr1. Saijo et al [25] showed that Nurr1 is SUMOylated by SUMO-2 and SUMO-3 in the lysine 558, in the context of an anti-inflammatory response in microglia. We replaced the lysine 558 by arginine (HA-Nurr1-K558R), and immunoblots of extracts from COS-7 cells transfected with this mutant showed the slow migrating 95-kDa band of similar intensity compared to wild type HA-Nurr1 immunoblots, indicating that lysine 558 is not a target of SUMOylation in our experimental conditions (data not shown). In addition, the truncated Nurr1 (HA-Nurr1ΔAF-1) did not show any slower migrating band in the presence of SUMO machinery, discarding that the LBD/AF-2 of Nurr1 are target of SUMOylation by SUMO-2. Therefore, we conclude that the SUMOylation of Nurr1 is principally occurring in the N-terminal region with lysine 91 as the main acceptor site of SUMO-2. It is worth to mention that nuclear receptor SUMOylation has been shown mainly in the N-terminal domain, for instance progesterone receptor [26], androgen, glucocorticoid and mineralocorticoid receptors [13].

Two pieces of information allow us to indicate that PIASγ behaves as the SUMO-E3 ligase of Nurr1. First, overexpressing PIASγ, but not PIASγmut1 that is unable to interact with Nurr1, significantly enhanced Nurr1 SUMOylation. Second, Nurr1 SUMOylation was no longer observed, when PIASγC342A, mutant that lacks SUMO ligase activity, was overexpressed. In addition, we suggest that PIASγ gives specificity and more efficiency to Nurr1 SUMOylation process, since a stronger and better defined band corresponding to Nurr1-SUMO-2 in immunoblots was observed in the presence of PIASγ. Our results allow us to propose that PIASγ exerts two inhibitory mechanisms over Nurr1 transcriptional activity. One mechanism requires its SUMO ligase activity. The SUMOylation of Nurr1 in the lysine 91 by PIASγ would limit Nurr1 transactivity in complex promoters (see below). Indeed, the mutant PIASγC342A exerted lesser repression than wild type PIASγ in reporter assays using complex promoters. The other repressive mechanism is independent of PIASγ SUMO ligase activity; because the mutant PIASγC342A exerted still a strong inhibition of Nurr1-dependent transcriptional activity, and we have shown previously [8] that the mutant Nurr1-K91R, which is resistant to SUMOylation is fully repressed by PIASγ. The immunofluorescent assays showing a similar strong colocalization of Nurr1-K91R with PIASγ compared to the wild type Nurr1, further support that PIASγ inhibition of Nurr1 is independent of its SUMOylation. Further work is required to reveal the mechanism by which PIASγ exert this dominant inhibition of Nurr1-dependent transcriptional activity and in what conditions only modulate its activity in the different type of promoters.

Data from literature indicates that SUMOylation can regulate several features of the transcription factors among them, the nuclear and/or subnuclear localization, stability and/or transcriptional activity [9] [11]–[12]. Our data suggest that SUMOylation of Nurr1 at lysine 91 does not modify its stability, since the SUMOylation-deficient Nurr1 mutant displays equal half-life than wild type Nurr1. Our half-life data for Nurr1 is similar to the half-life reported previously for Nurr1 [22]. The analysis of immunofluorescence data of Nurr1 and the mutant Nurr1-K91R and their colocalization with PIASγ indicates that there is no a relocation of the mutant K91R suggesting that SUMOylation does not regulate Nurr1 nuclear localization. Similarly, Belaguli and collaborators [27] showed that the SUMOylation of the transcription factor GATA4 did not modify its stability or the nuclear localization. Transcriptional repression induced by SUMOylation of transcription factors has been correlated with relocation of the transcription factor towards Promyelocytic Leukemia Protein bodies in the nucleus [28]. Additional work is required to learn whether the mechanism by which PIASγ represses Nurr1 is due to a relocation to silence sectors in cell nuclei.

Transcription factors interact among them giving synergic responses when they recognize multiple copies of their response element in the target promoters. This synergic regulation of transcriptional activity is restricted by SUMOylation of lysines within SC motifs present in some transcription factors [14] [29]. According with previous [8] and current results, SUMOylation decreases Nurr1 transcriptional activity. The lysine 91 of Nurr1, target of SUMO-2 is in a SC motif. This synergic control of transcriptional activity has been described for other nuclear receptors as progesterone [30], androgen [13], glucocorticoid [31] and estrogen-related receptors [32]. Nurr1 and Nurr1-K91R mutant showed similar transcriptional activity in the promoter with one NBRE element; however, Nurr1-K91R showed an enormous transcriptional activity compared with wild type Nurr1, when recognized three NBRE elements, demonstrating the classical behavior of transcriptional activity mediated by SC motif. Besides, Nurr1 has a glycine residue in position 85, preceding the SC motif core, further evidence of the presence of a functional SC motif [23] [29].

Our study provides evidence that Nurr1 SUMOylation controls its transcriptional synergy in complex promoters. Dopaminergic gene targets of Nurr1, such as tyrosine hydroxylase [33], RET [34] and dopamine transporter [35] harbor several NBRE elements combined with multiple elements for other transcription factors in their promoters. Future work is needed to reveal the regulatory role of Nurr1 SUMOylation in the control of the expression of genes of the dopaminergic system.
